# Hardship, coping, and joy: ACPs’ experiences of working through the COVID-19 pandemic

**DOI:** 10.1080/17482631.2025.2495382

**Published:** 2025-05-01

**Authors:** Emily Heavey, Melanie Rogers, Vanessa Taylor, Lihua Wu, Angela Windle

**Affiliations:** a Department of Social and Psychological Sciences, University of Huddersfield, Huddersfield, UK; b Department of Nursing, University of Huddersfield, Huddersfield, UK; c Department of Allied Nursing and Allied Health, Kingston University, London, UK

**Keywords:** COVID-19, advanced clinical practitioners, wellbeing, healthcare professionals, narrative

## Abstract

**Purpose:**

This paper reports Advanced Clinical Practitioners’ (ACPs) experiences of working in the United Kingdom during the COVID-19 pandemic, specifically the factors that impacted their mental, emotional, and physical wellbeing.

**Methods:**

The study presents qualitative data collected via two surveys in 2020 and 2021. Several survey questions elicited free-text responses, including a specific request for narratives. Narrative responses were thematically analysed and cross-referenced with non-narrative qualitative responses.

**Results:**

Three factors contributed to poor wellbeing: a changing work environment and expectations; bearing witness to the impact of Covid on patients; and the risk of catching and spreading Covid. Three factors improved wellbeing, whether through mitigating these challenges or directly, in the absence of specific hardship. These factors were new working practices; support structures; and individual resilience and self-managed coping strategies.

**Conclusion:**

This study expands research on professionals’ experiences of working through Covid to the under-researched experience of ACPs and demonstrates the intersecting and overlapping nature of factors contributing to poor and positive wellbeing. There are significant implications for stakeholders who need to consider the impact of future pandemics and opportunities for supporting and promoting wellbeing post-pandemic. The analysis also highlights the rich narrative data that can be collected using surveys.

## Introduction

The COVID-19 pandemic has been shown to have negatively impacted the mental health of healthcare workers across the world (Emirza & Kozcu, 2023; McEvoy et al., 2024; Nicolaou et al., 2021 ; Sasaki et al., 2020). In the United Kingdom (UK) specifically, research has demonstrated the high levels of psychological stress and burnout among healthcare staff during the pandemic (Petrella et al., 2021). While the negative impact on mental health was international, the experiences of individual healthcare workers were likely shaped by their own cultural contexts. In the UK, this context included caring for a patient population facing health and socioeconomic inequalities (Gogoi et al., 2023) and specific resource limitations within the National Health Service (NHS). Indeed, one UK study linked increased levels of anxiety and depression among healthcare professionals working in the pandemic to factors including insufficient personal protective equipment, workload, and workplace training and pandemic preparation (Gilleen et al., 2021).

This paper focuses on the experiences of an under-researched group of healthcare professionals in the UK: Advanced Clinical Practitioners (ACPs). ACPs are experienced clinical practitioners from various professions including nursing, pharmacy, and occupational therapy, and educated to master’s level. They differ from other non-medical healthcare professionals in relation to their level of practice and autonomy, use of expert knowledge, complex clinical decision making, and their ability to lead service provision that improves patient care (Kuczawski et al., 2024; Rogers et al., 2024; Rosa et al., 2020). ACPs operate at an advanced level in four pillars of practice: clinical, leadership and management, education, and research (Health Education England HEE, 2017). Their diversity of professional backgrounds enables ACPs to bring a wide range of skills and expertise to their roles (Diamond-Fox & Stone, 2021).

Similar to many healthcare practitioners in the UK and internationally, ACPs faced increased workloads, transitions to other areas of practice, and stress as a result of the pandemic. Previous research has shown how ACPs navigated multiple clinical challenges through the rapid transition in their duties and increased scope of practice (Morley et al., 2022). Their clinical expertise in management and leadership positions surpassed previous job limitations (HEE, 2020), as they utilized their advanced capability to make decisions, manage complexity and adapt to a constantly changing environment (National Health Service England, 2024). With the increasing need in clinical settings, ACPs recognized the areas in which their training and prior expertise required updating to align with their evolving responsibilities (Morley et al., 2022). However, many ACPs also reported feeling underutilized during the pandemic (HEE, 2020). Previous research has demonstrated that emotional and spiritual wellbeing and resilience among ACPs working during COVID-19 was low, although those with higher resilience demonstrated greater wellbeing (Rogers, Lamarche, et al., 2022; Rogers, Windle, et al., 2022). What remains under-researched is how working during the pandemic impacted the wellbeing of ACPs. In this study, we aimed to understand the factors that impacted wellbeing. In other words, what experiences positively and/or negatively influenced ACPs wellbeing and why? In this context, we define wellbeing as participants’ subjective experiences of psychological, emotional, and physical health and satisfaction. This draws on, but is not limited to, the concept of emotional wellbeing as defined by Feller et al. (2018). These authors use emotional wellbeing as an umbrella term covering various psychological dimensions, such as mood, emotion, mental health, life satisfaction, and sense of purpose.

This paper uses survey data from an exploratory study that focused on a cross-section of UK ACPs over a 2-month period in 2020 (Phase 1) and 2021 (Phase 2). The larger study was designed to determine participants’ emotional and spiritual wellbeing, and resilience in relation to their experiences during the COVID-19 pandemic. The current paper reports the qualitative findings collected from open responses to the surveys (both Phase 1 and Phase 2), with an emphasis on using the narrative data to identify the factors influencing the wellbeing of ACPs

### The value of stories

Storytelling is a way of constructing and conveying meaning in the recounting of events. When an individual tells a story of personal experience, they do not objectively list every event that happened in a certain period, but select, organize, evaluate and “frame” events, such that the story conveys their subjective views on, or experience of, those events (Polkinghorne, 1991; Riessman, 2008). Narratives make sense of experience, and particularly of unusual or unexpected experience (Bruner, 2002). Prototypical narratives are expected to include “trouble” (a disruptive or unusual event) followed by a response, in particular a “resolution” (Bruner, 1990; Labov & Waletzky, 1967). However, narratives need not be prototypical or focus on a single set of past events; they can also convey habitual or generic events (those which repeatedly occur), hypothetical or future events, and long stretches of time which to not focus on a single point or outcome (Riessman, 2008).

Narratives have been a consistent focus within healthcare research for the last several decades. Patients’ narratives can make sense of the disruptive experience of illness, help clinicians recognize unique experiences, and enable communication and advocacy with and for other patients (Frank, 2006; Greenhalgh & Hurwitz, 1999; Kleinman, 1988). Similarly, the narratives of clinicians and other healthcare professionals can provide insight into their experiences of working in the field and, as such, help researchers to understand complex experiences, events, processes, and flaws within that field (Greenhalgh, 2005; Wang & Geale, 2015).

Narrative research within healthcare research tends to rely on interview data. Narrative interview data have been used to explore healthcare workers’ experiences during the COVID-19 pandemic, including enacting new policies (Pilbeam et al., 2022), the practical and emotional aspects of providing care (Chung et al., 2021; Lapum et al., 2021), and constructions of resilience (Connolly et al., 2022; Hughes Spence et al., 2023). However, the collection of short, written narratives, for example through the elicitation of free-text responses on a survey, enables the researcher to collect large and diverse data sets while still retaining the richness that is so specific to the form (Winskell et al., 2018).

## Methods

### Data collection

In the larger study, an online survey was designed, comprising three validated scales assessing resilience, emotional and spiritual wellbeing (Rogers, Windle, et al., 2022). Eleven open questions were included in the surveys to elicit free-text responses (see Table I).

**Table I.** Survey questions eliciting free-text responses.

Q11	We are seeking stories of your personal and/or professional experiences as an ACP throughout the COVID-19 crisis. Please feel free to write a narrative in this section. This does not need to be formal or lengthy. Your thoughts, feelings and experiences are vital to help us understand how this pandemic has impacted you.
Q15	How is COVID-19 affecting your work and practice as an ACP/APN?
Q16	What coping strategies are you using to get through your shifts (hourly, daily and weekly)?
Q17	What things have been most helpful to you during this time?
Q18	How are you and your colleagues supporting each other?
Q19	What is giving you the strength to continue to work?
Q20	What are you doing to manage any stress you may be experiencing?
Q21	How has COVID-19 changed you as an ACP?
Q22	How do you believe the work and contributions made by ACP are being acknowledged or not?
Q23	What support do you feel you will need when the COVID-19 pandemic is over?
Q31	What support, if any, have you had during the COVID-19 crisis?

Snowball sampling was employed by asking recipients to share the survey link with colleagues and networks; as such, it is impossible to determine the number invited to participate. Additional invitations were sent to participants from Phase 1 of the study if they had shared their email address. All responses were anonymized. Inclusion criteria ensured participants were:

• Employed as an ACP and met the Health Education England (2017) definition of an advanced clinical practitioner: OR

• Credentialed as an advanced practitioner by either the Royal College of Nursing or a national government body OR

• Employed as a trainee ACP and were undertaking an advanced clinical practice master’s degree.

### Data analysis

A focus on narrative can help researchers to understand not only what happened, but why it was meaningful to a participant. Riessman (2008) notes that a focus on narrative can be usefully combined with more traditional qualitative research, by considering themes alongside structural and linguistic features of storytelling. Our analytic approach draws on thematic analytic techniques (Braun & Clarke, 2021) and thematic narrative analysis, which focuses on thematic content but keeps the story “intact” for analysis in order to build theory based on the narrative form rather than its themes alone. The narrative form is of value in understanding *why and how* participants’ wellbeing was impacted in the ways that it was.

Initial analysis focused on narrative responses to Q11 (“We are seeking stories of your personal and/or professional experiences as an ACP throughout the COVID-19 crisis … ”). Narrative data were identified as those that both conveyed sequential events and encoded the subjectivity of the respondent (Georgakopoulou & Goutsos, 2000; Riessman, 2008). Subjectivity was encoded if the text implicitly or explicitly conveyed the participant’s attitudes towards or evaluations of events, including through reporting past or present emotional responses.

Narrative data were analysed iteratively. The plot of each narrative was summarized, including its events and causality, subjective framing, and characters. These summaries created “data-driven” codes (Braun & Clarke, 2021) and acted as an “aide-mémoire” to allow more easy comparison of narrative plot arcs (Winskell et al., 2018). Codes were organized into themes and subthemes, specifically in relation to factors affecting wellbeing, with narrative “trouble” and “resolution” labelled if present. Narrative responses to the other qualitative questions were then identified and checked for consistency with these themes. Finally, the themes found within the narrative responses were cross-referenced with the non-narrative responses to all qualitative questions, to check for further examples, additional themes, and counter examples.

## Ethical considerations

Ethical approval was given by the University of Huddersfield. Prior to the study, participant information was provided and written consent was obtained. Data were protected under secure management at the principal investigator’s institution. All data were anonymized and accessed through secure password protected platforms.

Narrative data can carry a risk of sensitive disclosures, even in short written narratives of the sort we analysed. In face-to-face research, or research where the researcher has an ongoing relationship with the participant, situational or relational ethical choices can be made “in the moment”, in particular checking in with participants and seeking continuous consent (Ellis, 2007). Such techniques were not possible in the context of a survey study. However, participants were free not to answer any questions they found difficult or triggering. Care was taken to remove potentially identifying information such as names of patients or place names, even as stories themselves were kept intact. The distressing nature of the stories also carries a psychological risk for the researcher (Silverio et al., 2022). To mitigate this, the researcher who conducted the initial analysis has extensive experience of collecting and analysing qualitative data focused on potentially distressing topics including illness, hospitalization, and bereavement. The researcher received advice and guidance from the Principal Investigator about the nature of the data in advance of the analysis and held regular debriefings with the research team.

## Findings

In Phase 1, 734 respondents completed the survey. In Phase 2, 371 responses were received. Participants in both phases were aged between 25–67 years, with women being in the majority 88% (Phase 1) and 83% (Phase 2). Participants had practised as an ACP for between 1 and 24 years. Both phases included participants from Primary Care (55%), Secondary Care (22%), Intensive Care/Emergency Care (13%) and other settings (10%). Most participants were qualified ACPs (73%), the rest being trainee ACPs (27%).

Participants’ experiences of how working in the pandemic impacted their wellbeing varied greatly. Conflicting responses to the qualitative questions were common, as in these two non-narrative responses to the question “How has COVID-19 changed you as an ACP?”:

Remote assessment of patients which has probably increased my confidence overall. (Q21, 2021)

My self-esteem and confidence are at an all-time low. (Q21, 2020)

While some non-narrative responses indicated reasons for poor or positive wellbeing (as in the first response above), others did not (as in the second). However, the narrative data demonstrated common factors that contributed to poor or positive wellbeing.

Most narrative responses depicted “trouble”; that is, they described factors (events, actions, or other aspects of the pandemic) that had led to poor mental, emotional, or physical wellbeing. These factors are summarized in Table II.

**Table II.** Factors contributing to poor wellbeing.

Theme	Subthemes
Changing work environment and expectations	• New and changing regulations • High pressure and lack of support
Bearing witness to the impact of the pandemic on patients	• Witnessing Covid deaths and end-of-life isolation • Witnessing the impact of the pandemic on non-Covid patients
Risk of catching and spreading Covid	N/A

Many narrative responses depicted *only* these factors. In these narratives, working during in the pandemic was presented as painful, challenging, or otherwise problematic with no successful resolution to the difficult experiences described. Indeed, some explicitly expressed fears that the factors associated with poor wellbeing would continue or worsen in the future.

Conversely, other narrative responses described factors that led to positive wellbeing. These narratives often described the same “troublesome” factors shown in Table II, but also included events or actions that successfully mitigated or resolved that trouble. These resolutions could be framed as fragile, barely enabling the participant to cope with hardship, or as robust, anticipated to bring about longer-term wellbeing. Other narratives depicted no “trouble” at all, and only described events and actions that *directly* led to positive and joyful outcomes. The factors contributing to positive wellbeing—whether directly or through mitigating hardship—are shown in Table III.

**Table III.** Factors contributing to positive wellbeing.

Theme	Subthemes
New working practices	• Systemic practices and adaptations • Individual practices and adaptations
Support structures	• Colleagues • Patients and the public • Family and friends
Individual resilience and coping strategies	• Personal values and outlook •Self-managed coping strategies

No further overarching themes were found upon cross-checking the non-narrative data, suggesting that data saturation was reached within the narrative responses. The factors contributing to poor or positive wellbeing were broadly consistent across the 2020 and the 2021 survey responses.

Many survey responses reflected multiple themes. The following sections are organized for clarity, rather than to imply mutual exclusivity in the experience of the participant.

### Factors contributing to poor wellbeing

Working in the pandemic led to poor wellbeing for many participants. This included poor mental health, particularly anxiety and stress, distressing emotional responses such as sadness, guilt, anger, fear, and a sense of powerlessness or lost control. Poor physical responses were reported, especially exhaustion and, for some, Covid symptoms. The factors that led to these experiences are described in the following sections.

### Changing work environment and expectations

#### New and changing regulations

Working during Covid meant following radically different workplace regulations. This led to anxiety, stress, distress and, for some, a loss of control in relation to role. New regulations that were frequently mentioned as troublesome included the use of personal protective equipment (PPE), remote working and consultations, and a reduction in permitted patient visitors. These regulations were experienced by many participants as negatively impacting their own wellbeing, despite understanding the need for them.

Remote working could exacerbate the loneliness and isolation that many were already experiencing due to non-work-related restrictions, such as lockdowns, especially for those living alone:

I live on my own and so the majority of social contact would normally be at work. This experience has been very isolating and with no end in sight, I feel very despondent about the near future. (Q15, 2020)

Continuous wearing of PPE impacted on mental and physical health. Physical effects included dehydration and urinary tract infections due to long periods without drinking and dermatological problems such as dry skin and acne. One participant working in urgent care described the mental health effects experienced because of wearing PPE for long periods of time, and the subsequent impact on practice and sense of job satisfaction:

Every patient means wearing level 2 PPE for about 1.5 hours … I begin to panic as soon as I put the mask on too so I find I spend much less time with my patients than I used to and have to complete my notes sat in my response vehicle just so I can take the PPE off. It’s led to me questioning why I’m attending some incidents; it’s made me argue with colleagues about the most appropriate response, it’s making me miserable. And there is no end in sight for us. (Q11, 2020)

New regulations were introduced rapidly and were subject to change, particularly early in the pandemic. The lack of clarity around expectations led to anxiety, exhaustion, and a sense of helplessness and lost confidence. Confusion relating to where PPE should be worn and by whom was a common example. Participants also described feeling that they were inadequately trained for new duties which could lead to lack of confidence in their role.

#### High pressure and lack of support

This uncertain and constantly changing environment led to immense pressure. Staff worked long hours in conditions that felt unsafe, and many felt forced by unsupportive managers to work with inadequate protection. As an ACP working in the emergency department described it, *“we were like soldiers going to war but without the equipment we needed to ensure our safety”* (Q11, 2020). This combination of high pressure and lack of support led to anger, fear, stress, and “burnout”. A brief narrative illustrates several of these issues:

My role seeing patients in clinic changed to being on the Drs rota, working longer hours and seeing trauma and emergency patients. It has been totally demoralising that this has not been acknowledged by senior staff who only came into the hospital when needed. (Q11, 2021)

The participant’s sense of unfairness and lack of acknowledgement by absent management was a common theme, with several narratives presenting a divide between the frontline and management staff. As one participant commented as part of a longer narrative on the topic:

None of the nurse leaders were visible or supporting frontline staff during the pandemic they were hidden in their offices. They spent a lot of time praising each other but not recognising what the nurses on the frontline were actually going through. (Q11, 2021)

Participants also described feeling undervalued by patients and the public. Patients’ confusion, unrealistic expectations, or frustration about Covid regulations led to aggressive behaviour which, in turn, led to disillusionment for participants. One participant wrote, “*it is apparent that the clapping has well and truly ended and there seems to be a return of inappropriate use of services, media slating of the NHS and patient abusiveness which is incredibly disheartening”* (Q11, 2021). One ACP working in urgent care wrote a long narrative that described the experience of triaging a very ill patient, before describing the reaction of other patients to triage:

Other patients who were seen at front door triage had to be turned away to go home if they had covid symptoms and observations within normal parameters. One lady shouted at me that I was sending her home to die, it was hard for patients to understand. (Q11, 2021)

This sense of feeling undervalued and unsupported was also reflected in the many negative non-narrative responses to questions about support and acknowledgement participants had received (Q18, Q22, Q31).

### Bearing witness to the impact of the pandemic on patients

#### Witnessing Covid deaths and end-of-life isolation

Some of the most emotionally charged narratives related to participants’ experiences of bearing witness to Covid deaths. These experiences were repeatedly described using such language as “heartbreaking”, “haunting”, and “the hardest part” of the pandemic. Unexpected and rapid deterioration was particularly difficult, especially when a patient was young or previously healthy. These experiences invoked feelings of anger, helplessness, and shame when the participant felt that more might have been done. Other narratives simply described the exhaustion of witnessing such events again and again:

In one clinical shift … I was dispatched to three back-to-back prehospital cardiac arrests. Each patient had a history of COVID symptoms and each patient I stopped resuscitation and verified dead. I found myself saying a rehearsed speech to each family, almost without thinking what I was actually saying. I went home, to a house where I live on my own, feeling more tired (mentally and physically), than I have ever done in my career. I had a sense of dread knowing that the next day at work was going to be a repeat of that day. (Q11, 2021)

The physical isolation of patients from their families was a focus of many narratives of Covid deaths due to the Covid-related visitor restrictions impacting the ACP, the patient, and their family:

Watching a patient die from Covid, whilst her family listened to her animal like cries down the telephone was heartbreaking. The patients who were admitted for elective surgery immediately prior to Covid lockdown never saw their relatives again, dying a month later of their underlying cause whilst never having a visitor. Their final days will stay with me for the rest of my career. (Q11, 2020)

#### Witnessing the impact of the pandemic on non-Covid patients

Many responses described a reduction in non-Covid-related care due to new regulations, stretched resources, and the prioritization of the Covid response. Some ACPs viewed remote consultations as inadequate for assessing patients’ needs. This reduction in care caused participants to feel distress on the patients’ behalf, but also a loss of control, a sense of helplessness, anger, and anxiety about the invisible backlog of non-Covid illnesses that healthcare services would face in the future.

Delayed or reduced investigations, diagnoses, and treatment for various conditions were a particular concern. A short narrative from a colorectal cancer ACP illustrates the potential impact on participants’ emotional wellbeing.

I have found it very challenging to break bad news or tell a patient that they need cancer treatment but, due to Covid-19, they were unable to have the treatment. This has caused them more stress, upset & … I have often felt guilty, impotent & dis-empowered about this. (Q11, 2020)

Non-Covid patients’ mental health was also impacted by the pandemic as they faced isolation, fear, and financial hardships. This led to worry and stress for ACPs, and, in some cases, increased their workload as they were left to deal with these new mental health issues. In turn, this further increased the ACPs’ own emotional distress.

The patients I have seen in the GP surgery … . have become very low in mood, very isolated and it has been very difficult for them. This is what has impacted me, the loss to other people of their usual social interactions and life’s pleasures. One lady had not seen anybody for months and months, food was dropped at her door via online shopping organised by family, no conversation, no laughter, no cups of tea with anyone. Pure isolation at the grand old age of 90, it was heart-breaking for me. (Q11, 2021)

### Risk of catching and spreading covid

Whilst everyone is vulnerable to infection during a pandemic, narratives explicitly linked participants’ work with Covid patients to their own risk or experience of contracting the virus and spreading it to their families. The heightened risk engendered fear, anxiety, and anger. Participants who *had* contracted and potentially spread the virus experienced immense guilt.

Covid fears were heightened as participants witnessed the illness and deaths of patients and colleagues, and more so if they or their loved ones were clinically vulnerable. Some described taking precautionary steps which negatively impacted their wellbeing in other ways. An ACP working in gastroenterology described her experiences of fear early in the pandemic and the impact of her precautionary measures:

I remember the night the Prime Minister announced the national lockdown. I was so scared to go to work. Not for myself but because I didn’t want to bring the virus home to my husband and children. … . I became obsessed with hygiene. I was taking two showers a day and spraying my car down with antibacterial every journey. The skin on my hands was splitting from washing and gelling, my hair started to break off because I was over-washing it. (Q11, 2021)

Other participants chose to reduce physical contact with friends and family to reduce the risk of spreading Covid, but this led to feelings of isolation and loneliness. Some also expressed anger at their heightened risk, particularly when patients or colleagues were seen to increase that risk through negligence, for example hiding their own Covid status or taking unnecessary risks.

When participants did contract Covid, some narratives focussed on the distressing and exhausting sequelae. An ACP working in primary care described at length her symptoms, including a high fever lasting nearly two weeks, coughing, and vomiting. Three and a half months later, she was still experiencing lethargy, shortness of breath, and anxiety, taking a *“toll on [her] mental health*” (Q11, 2020). Other participants focused on the guilt they felt at having “brought Covid home” to their families. In one example, this guilt was compounded by the participant’s feeling that she had already caused her daughter’s anxiety due to her professional role, and then went on to spread the virus to her family: *“I felt very guilty about this … I brought home Covid and made them ill too. I will carry that forever”*. (Q11, 2020)

### Factors contributing to positive wellbeing

Despite the hardships associated with working in the pandemic, many responses referred to improvements in wellbeing. These included reducing negative responses such as anxiety, stress, and, fear, and increasing positive responses such as pride, a sense of control, confidence, and even joy. The factors that led to positive wellbeing, whether directly or through mitigating hardships, are discussed in the following sections.

### New working practices

#### Systemic practices and adaptations

While new and changing regulations could negatively impact participant wellbeing, many of the same regulations were cited as *improving* participant wellbeing. The difference was in how these changes were framed by participants in their narratives. Some suggested that new systems and adaptive ways of working had such a positive impact that they should become the norm for the NHS even as the pandemic died down.

Telephone triage and remote consultations, framed in the narratives presented in the previous section as isolating and potentially damaging to patient care, were presented in other narratives as an appropriate and effective way of addressing patient needs, leading to job satisfaction and reassurance for participants. For some, they were preferable to face-to-face consultations, allowing ACPs to feel more relaxed about workload, as in the following narrative.

Although our consultations are now predominantly telephone, I feel more relaxed and able to deal with my patient caseload. They are in the main much more appreciative and easier to manage as the time pressure has been lifted and I feel I can appropriately address their needs. I am actually doing more than I did pre Covid and when I see patients face-to-face I have a much better understanding of their concerns and expectations due to having been triaged prior to the appointment. This allows me to prepare my thoughts and formulate a plan. (Q11, 2020)

Working within systemic changes also meant the acquisition or improvement of skills and knowledge, including diagnostic skills. The need to learn new skills in rapidly changing roles and work without the usual support structures reminded participants of their adaptability and resilience. These experiences were a source of pride, confidence, and empowerment. An ACP working with older people described improving her skills and confidence:

Made me realise just how much I know and how clinically proficient [I am], because I had to rely on myself a lot with no-one to go to for help. Therefore, made me an even more confident practitioner. (Q11, 2021)

Some participants described being responsible for implementing changes, including workload allocation, infection control, and remote working practices. One narrative implicitly reflected the participant’s pride in their work, while also suggesting a positive impact on team wellbeing. The ACP’s long narrative described the disunity between teams within the primary care practice and their own leadership on a project to set up a new online consultation system, which led to “all members of all teams setting aside their differences and working together in a more cohesive, caring and inclusive way” (Q11, 2020).

Even socially distanced end-of-life care could instil feelings of pride alongside sadness. ACPs’ facilitation of remote connections with family members were framed in some narratives as a positive, safe alternative to physical contact (rather than a poor substitute), thus mitigating some of the distress associated with bearing witness to Covid deaths. An ACP working in a community hospital described their first experience of a Covid death in this way:

Our first COVID death however was very sad as she had initially been recovering well and suddenly deteriorated rapidly. Her daughter however was unable to visit as she was recovering from chemo and therefore shielding and her son lived further away. Holding the phone to her ear while they told her they loved her was very sad but we were proud as a team of the support we were able to give. (Q11, 2020)

### Individual practices and adaptations

For some participants, going to work was a source of wellbeing, as it could offer a sense of purpose and a distraction among the uncertainty and fear of living through a pandemic. An ACP working in primary care described in detail the various personal hardships the pandemic had brought about, including how she had struggled with feelings of isolation, concerns about the health of clinically vulnerable family members, and worries about the impact of the pandemic on her children’s education. The ACP concluded:

What I am trying to say is that throughout the whole pandemic and having to cope with things out of my control I have been grateful to go to work, have a job where I feel that I can support others (colleagues and patients) and feel that this has given me control in my life. (Q11, 2021)

Individual adaptations included acts of resistance: pushing back against new ways of working and “bending the rules” if necessary to provide comfort to colleagues and patients:

Another elderly lady was dying on the ward of a non-covid illness. She had twin sons who lived with her. We called her son who … . came in to sit with his mum while she was dying. He told me he had a twin brother … . He didn’t ask for his brother to be allowed in but I couldn’t imagine having to decide who gets to say goodbye … .so I told him we’d “bend the rules” and we let his twin brother come in. I thought it was important to do that. (Q11, 2021)

Such narratives conveyed a sense that the participant had done the right thing by their patients and can thus be understood as a strategy to improve their own wellbeing as well, through reducing the type of lingering professional distress that can come with bearing witness to patients suffering in isolation. However, these strategies were not without cost. Another participant described “always” holding the hands of dying patients, but noted “*we are however aware that this puts us at increased risk ourselves of contracting COVID”* (Q11, 2020).

Protecting oneself from work-related hardships by stepping away from work was another individual strategy to improve wellbeing. For some, this meant taking leave. However, many responses described leaving or planning to leave their role to protect their wellbeing.

### Support structures

#### Colleagues

Interacting with colleagues helped participants to reduce the isolation of the pandemic, giving a sense of normality and improving mental health. This protective effect is illustrated here:

I struggled with the social isolation, and found my mental health fell sharply with severe depression and suicidal ideation. Work became a place of focus, comfort and purpose providing the ability to work escape these. Colleagues and peers became my sole focus of interaction and my entire driver for not killing myself. (Q11, 2021)

Colleagues could also be sources of emotional support and comfort in response to specific hardships at work. One participant wrote about a period of redeployment to the intensive care unit; the understaffing in the unit and her own lack of experience in the role were challenging, while the long shifts in PPE were exhausting. She went on to describe how speaking with colleagues who understood her experiences was a comfort.:

I am normally very good at leaving my job at work, however during this time on ITU I could think of nothing else - it totally consumed my thoughts and I didn’t sleep very well. I was offered virtual support sessions on Zoom with colleagues who were doing the same thing and found these really useful. (Q11, 2021)

Several responses referred to the usefulness of such organized support sessions with colleagues, including scheduled drop-in sessions, “wobble rooms” where staff could bond and reflect, debriefs after challenging events, and workplace counselling sessions. Such responses highlighted not only peer support, but the support *structures* put in place by management. Other responses emphasized informal support between colleagues, including social media messaging groups, chatting and joking, and out of work socializing where permitted.

#### Patients and the public

While some patient interactions contributed to poor wellbeing, others improved it; participants described expressions of gratitude for good care or acknowledgement of the challenges they faced as reducing distress and helping them to feel proud and even joyful. An ACP working in primary care wrote about treating a patient after a telephone assessment:

I worried all week in case I’d missed something. The following week he was on my telephone consultation list. My heart sank. It turned out he wanted to thank me personally for taking the time to thoroughly assess, as much as I could on the phone … .this feedback came on a day I was feeling quite stressed… I went home feeling much better, mentally. (Q11, 2020)

Members of the public were also a source of support and comfort outside the workplace. Many participants mentioned the renewed public appreciation for healthcare staff while others cited individual supportive encounters that helped them to cope, as in the following example.

One night I was driving home from work and I found myself crying at the traffic lights. The thoughts of what was to come overwhelming. People were carrying on their daily activities without any idea of what was going to happen. I was worried for my family, friends, colleagues and patients. I looked to my right and there was a young lad looking at me obviously he had seen me crying. He mouthed “are you ok?” I nodded and said “yes thank you.” He smiled and carried on. That act of human kindness gave me the will to get on with it and pull myself together. (Q11, 2020)

#### Family and friends

While family and friends could not offer support in the physical context of the workplace, several narratives mentioned returning home to these support structures as a source of relief from the pressures and distress associated with working in the pandemic. An ACP described a combination of challenges, including the grief of the families she worked with and fears around her own family contracting Covid. Her family and friends were a great source of support in facing these hardships:

All this has been extremely challenging and yet we have pulled together as a family and supported one another. We have had prayer and kind words from our friends and feel hopeful that we have made the right choice and that this isolation will come to an end before harm is done emotionally (Q11, 2020)

Family and friends were very commonly cited in responses to questions about what had helped participants cope, manage stress, and stay strong (Q16–17, Q19–20) and what support they had (Q31). Participants referred to spending time with loved ones, but also thinking about going home to them during the trials of the working day.

### Individual resilience and coping strategies

#### Personal values and outlook

Many participants described their personal values and outlook as resources that helped them to cope or even thrive. This could relate to spirituality, faith, being resilient or having a positive attitude or a strong work ethic. For some, such values were long held, rather than specifically triggered by the pandemic. This was the case with a participant whose narrative described how resilience and faith enabled her to cope:

It was quite daunting to be re-deployed in an unfamiliar environment, looking after unresponsive critically ill patients and the uncertainty about Covid and whether I could easily catch it and die. I was stressed but I have always been resilient and talked myself through what my calling was and put my faith in God (Q11, 2020)

While some presented such values and attitudes as innate, for other participants a resilient outlook was a necessity of the pandemic, something that they had to cultivate or enforce within themselves. Some described “compartmentalising” the difficulties of working life, forcing themselves not to overthink, or deliberately focussing on colleagues, patients, or the fact that they were getting paid. One participant described myriad challenges, including sparse and inaccurate information, breaking bad news over the phone, not being able to attend to patients without correct PPE, and having to prevent relatives from visiting patients. She went on to describe the emotional distress of colleagues and patients, and how she coped:

Staff were terrified and some refused to work initially because they were so afraid. Not being able to hug people has been very difficult because it has been a very emotional time for staff, patients and relatives … .I cried a lot over the first weekend but as soon as I was able to compartmentalise things and provide solutions to some of the problems that arose initially I was able to cope. (Q11, 2020)

The passing of time helped some participants to become more accepting and adopt a more resilient attitude. This was particularly so as knowledge about the virus improved, as vaccines became available, and as participants or those they knew contracted Covid and recovered.

#### Self-managed coping strategies

Other participants drew resilience from self-managed coping strategies. These strategies were usually listed as brief, non-narrative responses, particularly in response to Q16–17 and Q19–20, although they also appeared in a few narratives.

Relaxation techniques were a common strategy, particularly yoga, mindfulness, and meditation. An ACP working in primary care described how such techniques helped her manage guilt around working from home and anxiety around catching and spreading Covid:

I felt emotional most of the time, I was aware of carrying tension and anxiety in my shoulders and I had problems relaxing and sleeping. My sleep was often fitful and full of vivid dreams - yoga and mindfulness helped me to manage these better over time. (Q11, 2020)

Other common self-managed coping strategies were exercise, in particular walking and running; healthy eating; and taking time for hobbies such as music, watching television, reading, and crafts. Some participants also mentioned avoiding news and other media to help them “switch off” from the pandemic when not at work. A few mentioned making use of mental health interventions such as medication or therapy outside the context of work-provided support sessions.

Several non-narrative responses cited less healthy coping strategies, which did not appear in the narrative responses. These included smoking, drinking more alcohol, and comfort eating. These were not mutually exclusive with the healthier strategies, as demonstrated in the following brief example:

Meditation, honestly alcohol. (Q20, 2021)

## Discussion

In this research, our aim was to understand the factors that impacted the wellbeing of ACPs in the UK during the COVID-19 pandemic, including what experiences positively and/or negatively influenced ACPs wellbeing and why. Three factors were identified as negatively affecting ACPs’ wellbeing, in particular, their psychological and emotional wellbeing. The changing work environment, bearing witness to the impact of the pandemic on patients, and the risk of catching and spreading Covid could all lead to anxiety, stress, fear, anger, sadness, guilt, lost confidence, powerlessness, and loneliness. Less commonly described were negative effects on physical wellbeing, including Covid symptoms, exhaustion, and various physical impacts of hygiene measures. Three factors contributed to positive wellbeing experiences. These were new working practices, support structures, and individual resilience and coping strategies. These factors helped participants to cope by mitigating poor wellbeing or the factors that caused it. They could also directly lead to specific positive forms of wellbeing, such as pride, enthusiasm, and confidence, which we will collectively refer to as “joy”.

Our findings about ACPs’ experiences both support and extend previous findings relating to understanding healthcare workers’ experiences of working during the pandemic. Additionally, this research contributes a longitudinal perspective to the evidence to illustrate trends over time and the collection of in-depth data from this group. Previous studies have highlighted the negative impact of working during the early phase of the pandemic on healthcare workers’ mental health (Aughterson et al., 2021; Hoernke et al., 2021; Nyashanu et al., 2020). Similarly, for ACPs early in the pandemic during phase 1 of our research, the experiences of ACPs illustrate the links between the ACP role, working conditions and resources, and personal wellbeing. The ACPs were concerned about risks to themselves and others from insufficient resources including PPE, COVID testing, staffing and guidelines. Aligned to other studies (Dunn et al., 2020; MacDougall et al 2021), the ACP’s acknowledged their duty of care, but raised concerns about adequate resources, staffing and appropriate preparation for redeployment to enable them to deliver care safely and well. Our study also illustrates how changes to the ACPs’ professional role, and structural and procedural changes to care provision with a lack of managerial support and engagement all contributed negatively to their wellbeing in the early part of the pandemic and, for many, accumulated over time as the pandemic progressed. This has led to concerns about the retention of this experienced workforce group following the pandemic.

Other authors have similarly reported that a lack of clarity around evidence, COVID-19 guidelines and policies negatively impacted healthcare workers’ experiences of working in the pandemic (Lapum et al., 2021), and specifically how this can lead to a greater workload and high-pressure working environment (Pilbeam et al., 2022). The helplessness, pain, and anger associated with witnessing Covid deaths and the isolation patients experienced have also been highlighted (Lapum et al., 2021), alongside the frustration associated with seeing the Covid response take precedence over other forms of care (Pilbeam et al., 2022). Indeed, during the early phase of the pandemic, ACPs were concerned about the changes to usual care, particularly for people with chronic or life-limiting conditions. As the pandemic progressed, however, and recognizing the potential damage from delays to non-COVID care, some ACPs transformed their ways of working, using the opportunity for professional development and positive impact to deliver innovation in their service delivery leading to pride, enthusiasm, and confidence and enhanced wellbeing.

Regarding factors contributing to positive wellbeing, Pilbeam et al. (2022) showed that healthcare workers adopted individualized solutions to certain challenges, such as seeing patients strategically to preserve PPE or choosing not to socially distance when breaking bad news. By contrast, Ding et al. (2022) identified “giving up” as a rare strategy for coping among frontline healthcare workers. Our findings, while not quantitative, identified many instances of participants citing leaving their jobs as a possible individual adaptation to working practices. The importance of collegiate support has been highlighted in relation to resilience both pre-Covid (Cameron & Brownie, 2010; Jackson et al., 2007) and during the pandemic (Chung et al., 2021; Connolly et al., 2022). Ding et al. (2022) and Fournier et al. (2024) observed the importance of friends and family as sources of support, while (Chung et al., 2021) highlighted the importance of gratitude from the public. Various studies have observed the importance of personal values and outlook, including resilience tied to professional identity (Connolly et al., 2022), positivity (Ding et al., 2022), containing emotions (Lapum et al., 2021), and greater acceptance with greater knowledge of the disease (Chung et al., 2021). Ding et al. (2022) note engagement with mass media as a common self-managed coping strategy; while watching television and listening to music were cited by some of our participants, some also cited *avoiding* certain forms of mass media such as the news and social media.

Alongside extending these earlier findings to the under-researched longitudinal experience of ACPs, this study is novel in in that it particularly highlights the intersecting, overlapping, and at times apparently contradictory nature of factors contributing to poor and positive wellbeing. Inevitably, participants could experience multiple factors simultaneously, but moreover, the factors described above could influence each other in various ways. For example, bearing witness to patients dying of Covid increased participants’ fears of contracting and spreading Covid, while aspects of the changing working environment such as remote consultations could lead to witnessing reduced care for non-Covid patients, which in turn could lead to a lack of support *from* those patients. The same is true of factors contributing to positive wellbeing. For example, the support and gratitude of patients could fuel personal values such as work ethic, while many systemic adaptations and actions were facilitated by the support of colleagues. Some factors also had the potential to cause new problems; for instance, breaching social distancing rules to comfort patients reduced patient and participant distress in the moment, but could heighten participants’ fears of their own risk of Covid.

**Figure 1.**
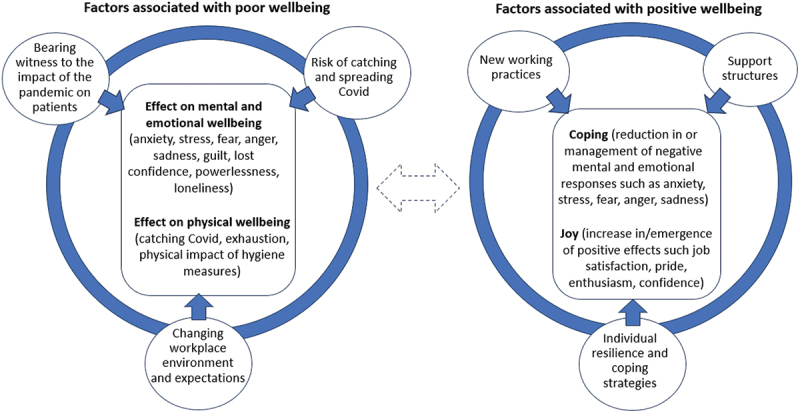
Factors associated with poor and positive wellbeing.

The interlinked and overlapping nature of factors that could contribute to poor and positive wellbeing are illustrated in Figure 1. The dashed double arrow between the two groups of factors indicates the potential (but not inevitable) movement between them. A participant experiencing poor wellbeing might move towards factors associated with positive wellbeing to cope or find joy; yet solutions may be fragile, failing to sustain positive wellbeing, or may even lead to new forms of poor wellbeing.

While there was thematic consistency across the data, causative connections are also identified: specifically, how particular events led to impacts on wellbeing. This included demonstrating the contrast between those experiences of hardship that were resolved (or at least mitigated) and those that were not. In narrative terms, some stories presented “trouble” followed by “resolution” and some presented only trouble (Bruner, 1990; Labov & Waletzky, 1967). The narrative data also demonstrated how events, actions, and situations related to working in the pandemic could lead to different impacts for different participants. For example, regulations such as remote working could *contribute* to poor wellbeing for one participant and could *mitigate* poor wellbeing for another. The narrative data also demonstrated the potential for fragility or robustness of strategies, and the possibility that in solving one crisis, another may emerge. While narratives have long been valued in health research, such research commonly relies on spoken narratives obtained through interviews. This article adds to the literature suggesting the value of written narratives—even very brief ones—for understanding complex and conflicting experiences (Winskell et al., 2018). Compared to interview data, collecting free-text survey responses can be more easily done at scale, is less time-consuming for participants, and allows a greater sense of privacy and anonymity for those sharing difficult experiences, while still retaining the richness associated with narrative data.

## Limitations

The study has several limitations, in particular, in relation to the sample and the data collection. ACPs are not regulated in the UK, therefore we relied on participants self-determining if they met the inclusion criteria. Relatedly, the numbers of ACPs working in the UK in unknown so we may not have reached all eligible participants. A large majority of our participants were women; given that the gender breakdown of ACPs nationally is unknown, our sample may or may not be proportionally representative in this regard. In either case, the study is limited in its representation of the experiences of men and non-binary people working as ACPs in primary, community and secondary care.

Despite the potential utility of narrative data collected via surveys, the method has clear limitations. Researchers are unable to probe, encourage, or clarify participants’ responses, such that these data may lack the depth and richness of data gathered face-to-face (or even via telephone). In particular, it was not possible to prompt exploration of how participants’ individual circumstances, including age, ethnicity, gender, and family situation, may have impacted their experiences. Finally, the fact that many participants gave very brief, non-narrative responses in response to the question eliciting narratives (Q11), or no responses at all, suggests that some people may be unwilling or unable to share their narratives via this method of data collection.

## Implications for practice

The pandemic increased pressure on an already strained healthcare system, significantly impacting on the wellbeing of ACPs. This study highlights key factors with the potential to negatively or positively impact the wellbeing of ACPs working during the Covid pandemic. These findings may be applicable to other groups of healthcare professionals working in other pandemics, other health crises, or, indeed, in the event of a re-emergence of the Covid pandemic. Recognizing factors contributing to positive wellbeing is vital, so that these factors may be supported, for example by enabling greater access to forms of peer support where desired, establishing and embedding the kinds of systemic changes that support wellbeing, and supporting ACPs with self-managed coping strategies where appropriate. In addition, using the expertise of ACPs and, for some, facilitating innovation and new ways of working in their practice and service delivery promoted wellbeing and enduring benefit. It is also useful to recognize the types of unhealthy coping strategies that ACPs may rely on, so that they can be supported to access healthier strategies if they wish. More research is needed into the long-term effects of certain coping strategies, particularly those which appear fragile or may place a significant mental burden on ACPs, such as “compartmentalizing” or adopting a “just get on with it” attitude.

Recognizing the factors that contribute to poor wellbeing is similarly vital. Where possible, these should be addressed and mitigated; for example, clarity of role and supportive management structures could mitigate the poor wellbeing associated with rapid change and a high-pressure environment. It is also important to recognize that not all factors contributing to poor wellbeing can be prevented; for example, witnessing Covid deaths is a sad inevitability of working during the pandemic. In these cases, enabling factors contributing to positive wellbeing is particularly crucial. Finally, there is value in simply acknowledging ACPs’ stories of potentially unfixable (or escalating) hardships, which have parallels with what Frank (1995) calls “chaos narratives”. Chaos narratives are stories of illness that do not imagine a cure but instead present continuing and unfixable hardships. These stories provoke anxiety in an audience, and perhaps particularly in clinicians because “chaos is an implicit critique of the modernist assumptions of clinical work” (Frank, 1995, p. 111). However, recognizing these types of stories—and having them recognized—can be crucial for the audience and the teller. They are sources of learning and empathy for the audience and potentially necessary outlets for the teller. As Frank (1995, p. 110) puts it, “‘getting out of chaos is to be desired, but people can only be helped out when those who care are first willing to become witnesses to the story”.

Covid-19, and the response to it, has profoundly impacted ACPs in ways which must be acknowledged and addressed by employers. In this study, we have focused on ACPs, an under-researched workforce, and the impact and radical changes they experienced during the COVID-19 response. For these practitioners in the post-pandemic recovery phase, learning and actions by stakeholders at national/system, organizational, managerial, team and individual levels include recognizing the importance of not returning to business as usual without considering the long-term psychological needs of the workforce (British Psychological Society, 2020; Pollitt & Pow, 2022). The British Psychological Society (2020) and research from The Policy Institute (Pollitt & Pow, 2022) offer recommendations for how to respond for staff in the “recovery” phase and for restoring and maintaining staff wellbeing for the future. The NHS Check project aims to use the evidence it generates to better support the future needs of NHS staff by informing workforce planning, targeted support for individuals, and more effective strategies for emergency response. The recommendations include the principles and actions to support the wellbeing of staff as “getting the basics right”, “creating the right culture” and “learning and planning” aiming to reorientate staff support from a model of detecting and treating disorder, to one where prevention is key and a focus on good mental health for all (Pollitt & Pow, 2022).

## Data Availability

The participants of this study did not give written consent for their data to be shared publicly, so due to the sensitive nature of the research supporting data is not available.
